# Multiple Modes of Action of a Monoclonal Antibody against Multidrug-Resistant Escherichia coli Sequence Type 131-*H*30

**DOI:** 10.1128/AAC.01428-17

**Published:** 2017-10-24

**Authors:** Luis M. Guachalla, Katharina Hartl, Cecília Varga, Lukas Stulik, Irina Mirkina, Stefan Malafa, Eszter Nagy, Gábor Nagy, Valéria Szijártó

**Affiliations:** Arsanis Biosciences GmbH, Vienna, Austria

**Keywords:** Escherichia coli, monoclonal antibody, ST131, complement-mediated killing, endotoxin neutralization, mechanism of action, opsonization

## Abstract

The multidrug-resistant *H*30 subclone of extraintestinal pathogenic Escherichia coli sequence type 131 (ST131-*H*30) has spread worldwide. This clone expresses a conserved lipopolysaccharide (LPS) O antigen, O25b. Previously, we described monoclonal antibodies (MAbs) specific to the O25b antigen and characterized them as diagnostic and therapeutic tools. In this study, evidence is provided that besides the previously shown complement-mediated bactericidal effect, an O25b-specific humanized MAb, A1124, also enhances opsonophagocytic uptake by the murine macrophage cell line RAW 264.7. Both phagocyte-dependent killing and phagocyte-independent killing, triggered by A1124, were confirmed in human whole blood. Furthermore, A1124 was shown to neutralize endotoxin activity of purified LPS of clinical isolates. This activity was demonstrated *in vitro* using both RAW 264.7 cells and a human Toll-like receptor 4 (TLR4) reporter cell line, as well as in a murine model of endotoxemia using purified LPS for challenge. Significant protective efficacy of A1124 at low doses (<1 mg/kg of body weight) was shown in murine and rat models of bacteremia. The contribution of the bactericidal and anti-inflammatory effects was dissected in the mouse bacteremia model through depletion of complement with cobra venom factor (CVF). Protective efficacy was lost in complement-depleted mice, suggesting the essential role of complement-mediated activities for protection in this model. These data suggest that A1124 exhibits different mechanisms of action, namely, direct complement-mediated and opsonophagocytic killing as well as endotoxin neutralization in various challenge models. Which of these activities are the most relevant in a clinical setting will need to be addressed by future translational studies.

## INTRODUCTION

Extraintestinal pathogenic Escherichia coli (ExPEC) is a common pathogen responsible for urinary tract and intra-abdominal infections, pneumonia, bacteremia, meningitis, and other invasive infections. During the second half of the last century, most of these infections could be successfully treated with antibiotics. The extensive use of antibiotics, however, has selected for drug-resistant variants that often accumulate resistance factors to multiple classes of antibiotics. The emergence of such multidrug-resistant (MDR) strains is partly a clonal phenomenon, although only a few successful clonal lineages combine resistance with retained fitness and virulence (1, 2). One of the most-investigated MDR clones, subclone *H*30 of E. coli sequence type 131 (ST131-*H*30), has spread globally, in both nosocomial and community settings (3–5), since its first description less than a decade ago (6, 7). Nowadays, ST131-*H*30 alone is responsible for 10 to 25% of all extraintestinal E. coli infections (8–10). Among MDR strains, its prevalence is even higher. This lineage has reached absolute dominance (i.e., >50%) among quinolone-resistant (11, 12) as well as extended-spectrum beta-lactamase (ESBL)-producing isolates (13). The progressive acquisition of additional resistance traits in ST131-*H*30 strains is alarming. Resistance of E. coli to last-resort drugs, such as carbapenems and colistin, is emerging, and ST131 isolates often predominate among such isolates (14–17). This forecasts the emergence of pan-resistant E. coli strains, which poses a great threat given the slow pace of development of novel antibiotics against Gram-negative pathogens (18).

In search of alternative therapeutic approaches (19), monoclonal antibodies (MAbs) were developed (20–23), which offer several potential advantages over other biologics such as polyclonal sera, antimicrobial peptides, and bacteriophages. Human and humanized MAbs are not expected to elicit an immune response and are not projected to have an impact on the normal microbiome due to their precision targeting.

We previously reported that the unique lipopolysaccharide (LPS) O antigen O25b, which is conserved in the ST131-*H*30 clonal lineage, represents an attractive molecular target that is accessible through various capsular polysaccharides. Due to this, O25b-specific MAbs were shown to be suitable for diagnostic purposes (24); furthermore, evidence was provided for their prophylactic efficacy (25). In this study, we corroborate a high protective efficacy of a selected humanized O25b MAb (A1124) in various rodent models. Furthermore, we describe three distinct mechanisms of action for this single MAb that were distinguished by various *in vitro* assays and in murine models.

## RESULTS

### Antibody generation.

Murine MAbs were generated against the O25b antigen by hybridoma technology as described previously in detail (24). The murine MAbs were humanized by grafting the complementarity-determining region (CDR) into the closest human IgG1 heavy-chain and kappa light-chain framework sequences. Specificity and binding characteristics of the humanized offspring of several murine MAbs were confirmed and reported earlier (25). The MAb A1124 used in this study is a sibling (i.e., sharing CDRs but having different framework sequences) of the previously described 3E9-11 MAb (25).

### Complement-mediated killing.

**FIG 1:**
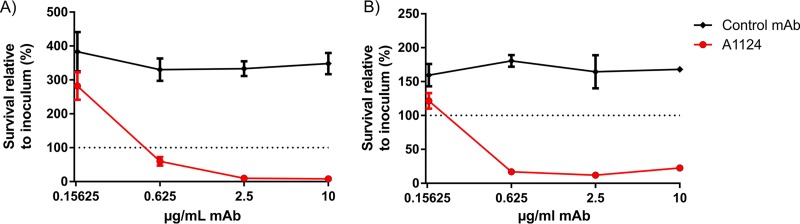
Complement-mediated bacterial killing triggered by A1124. E. coli ST131 strains (81009 [A] and 3O [B]) were incubated in 50% human serum (depleted with the corresponding strain) in the presence of the indicated doses of MAbs. The recovered CFU was related to the input bacterial number. Graphs show the mean ± standard error of the mean obtained from 3 (A) and 2 (B) independent experiments.

Antibody-dependent complement-mediated bactericidal activity of MAb A1124 was measured in a serum bactericidal assay (SBA). Bacteria were incubated in human serum samples that had been depleted of ST131-specific antibodies, in the presence of different concentrations of A1124 or an isotype control IgG with irrelevant specificity. A dose-dependent bactericidal activity was observed with maximal effect starting at doses as low as 0.625 μg/ml (Fig. 1). Complement dependency was confirmed by using heat-inactivated (i.e., complement-inactivated) or cobra venom factor (CVF)-treated (C3 depleted by consumption) serum samples, in which a net bacterial growth was observed irrespective of the presence of any MAbs (data not shown).

### Opsonophagocytic uptake.

**FIG 2:**
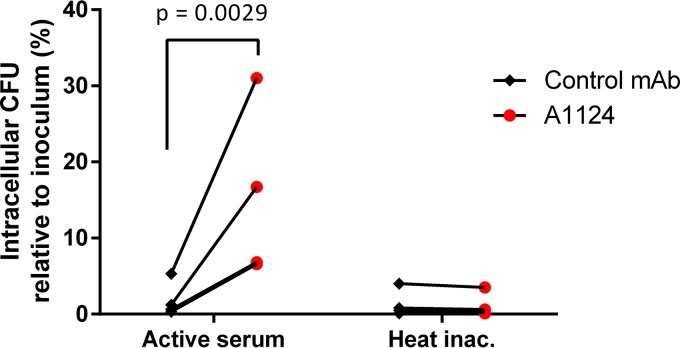
Opsonophagocytic uptake of bacteria induced by A1124. E. coli ST131 cells were preincubated with 2.5 μg/ml antibody in 5% adsorbed human serum with or without heat treatment. Preopsonized bacteria were incubated with RAW 264.7 murine macrophage cells at an MOI of 1 for 1 h, and uptake was measured by eliminating extracellular bacteria with 40 μg/ml kanamycin for an additional 2 h. Graphs show results of 4 experiments. Statistical analysis was performed using the ratio paired *t* test.

Opsonization of E. coli ST131 by MAb A1124 was tested using the murine macrophage cell-line RAW 264.7. Bacteria were incubated with the phagocytes at a multiplicity of infection (MOI) of 1 in the presence of A1124 or an isotype control MAb, and the intracellular CFU was determined following elimination of extracellular bacteria (Fig. 2). Baseline uptake, which was independent of the complement, was <5% of the original inoculum. A1124, but not the control MAb, significantly increased bacterial uptake (12.8 ± 2.9-fold). Inactivation of the complement abolished the MAb-dependent uptake of this strain, suggesting a complement receptor (CR)-mediated process.

### Bactericidal activity in human blood.

Bactericidal activity of A1124 was measured in a human whole-blood assay. To distinguish between cell-mediated (phagocytosis) and humoral bacterial killing, the actin polymerization inhibitor cytochalasin D (CytD) was employed to inhibit phagocytosis. In pilot studies, CytD was confirmed not to have any influence on phagocyte viability or complement activity (data not shown).

**FIG 3:**
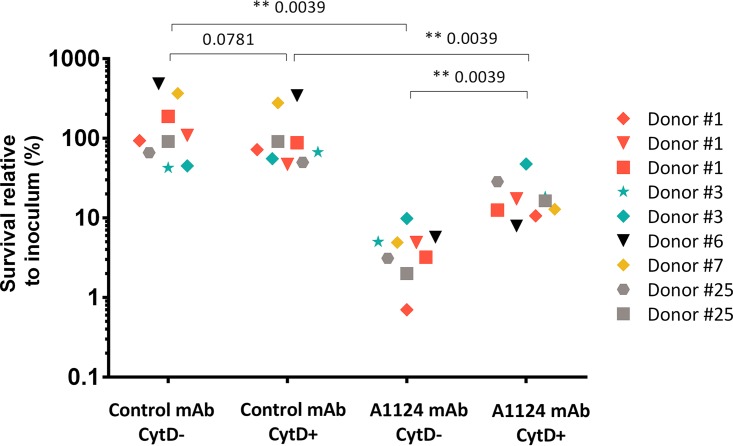
Dissection of phagocyte-dependent and -independent bactericidal activities of A1124 in human blood. Bacteria were added to heparinized blood pretreated with either cytochalasin D (CytD+) or its buffer as control (CytD-) and test antibodies and incubated for 3 h. Surviving bacterial count was correlated with the input number of bacteria. Data from 9 independent experiments using blood samples of 5 different donors are shown. Statistical analysis was performed using the Wilcoxon matched-pair signed-rank test, and *P* values are indicated on the graph.

Survival rates of E. coli ST131 opsonized with control antibody greatly differed in fresh human whole blood obtained from different donors but showed good reproducibility for repeated blood samples—drawn up to three times—from the same donor (Fig. 3). CytD treatment did not elicit an appreciable effect on bacterial survival (see Fig. S1 in the supplemental material). Preopsonization of bacteria with A1124 resulted in significantly reduced survival in all blood samples in both the absence and the presence of CytD. Importantly, inhibition of phagocytosis significantly increased bacterial survival in the blood of four out of five donors, demonstrating that in addition to its complement-mediated bactericidal activity, A1124 also elicits opsonophagocytic killing activity (Fig. 3 and S2).

### Neutralization of endotoxin activity.

**FIG 4:**
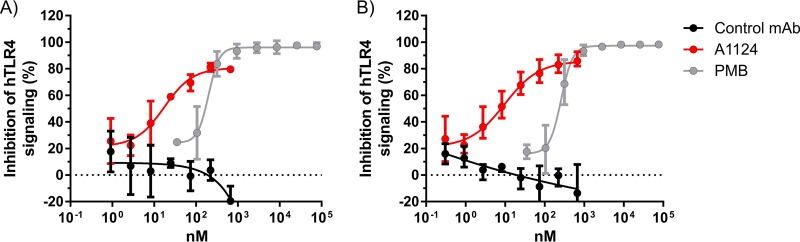
Inhibition of hTLR4 signaling by MAb A1124. Effect of A1124 on LPS signaling through hTLR4 was measured with a commercial reporter cell line system (HEK-Blue). Antibody-dependent inhibition of TLR4 signaling induced by purified LPS from ST131 strain 81009. (A) No serum added. (B) In the presence of 4% human serum pool. In all experiments, polymyxin B (PMB) and an isotype-matched MAb with irrelevant specificity served as positive and negative controls, respectively. Graphs show combined results of 2 (A) and 3 (B) experiments, indicating mean ± range.

The target of A1124, namely, the O25b antigen, is part of the lipopolysaccharide molecule that exerts endotoxin activity through its lipid A portion. In order to investigate whether binding of A1124 to O25b LPS interferes with the signaling through human Toll-like receptor 4 (hTLR4), a commercial *in vitro* colorimetric assay was applied. HEK293 cells expressing the human TLR4 receptor complex were incubated with LPS purified from ST131 E. coli (the optimal LPS concentration range for hTLR4 activation was determined in pilot studies [data not shown]). In the presence of A1124, a dose-dependent inhibition of hTLR4 signaling was observed (Fig. 4A). Importantly, the potency of A1124 was approximately 10-fold higher than that of polymyxin B, a small-molecule antibiotic with known endotoxin-binding activity.

As therapeutic MAbs are intended for parenteral application and LPS is known to interact with serum proteins such as lipopolysaccharide-binding protein (LBP), albumin, bactericidal permeability-increasing protein (BPI), etc., we aimed to clarify whether human serum would interfere with the endotoxin-neutralizing activity of A1124. Therefore, the same assay was repeated in the presence of normal human serum. We found that the presence of serum (4%) did not affect the inhibition of hTLR4 signaling by A1124 (Fig. 4B).

**FIG 5:**
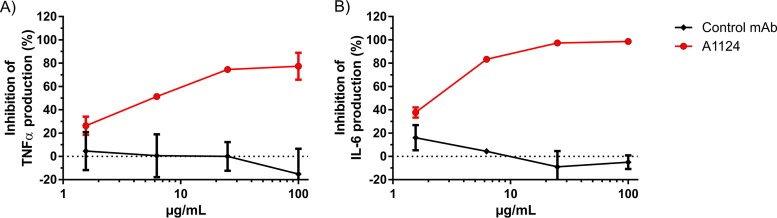
Inhibition of the LPS-induced inflammatory cytokine release of cultured macrophages by A1124. RAW 264.7 cells were incubated in the presence of MAb and subsequently stimulated with 10 ng/ml of LPS extracted from E. coli ST131 strain 81009. Supernatants were collected 24 h after the addition of LPS, and the amount of cytokines (TNF-α [A] and IL-6 [B]) was determined by ELISA. Mean ± range of the percent inhibition from 2 independent experiments is shown.

Cytokine release of cultured macrophages was measured to investigate the impact of blocking LPS-induced hTLR4 signaling by A1124. RAW 264.7 cells were stimulated with purified LPS in the presence of A1124 or an irrelevant control MAb, and the released inflammatory cytokines from the culture supernatants were measured. A1124 potently inhibited the production of both tumor necrosis factor alpha (TNF-α) (Fig. 5A) and interleukin-6 (IL-6) (Fig. 5B) in a dose-dependent manner.

### Endotoxin neutralization *in vivo*.

**FIG 6:**
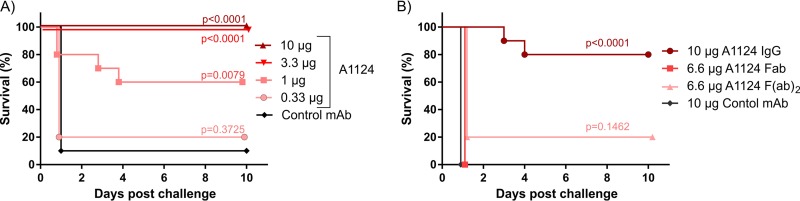
Protective efficacy of A1124 in a murine model of endotoxemia. Groups of 5 BALB/cJRj mice were passively immunized with various concentrations of A1124 IgG (A) or equimolar amounts of IgG and its fragments with respect to antigen-binding sites (B). Challenge with a lethal dose (2 ng) of purified ST131 LPS was performed in GalN-sensitized mice 24 h (A) or 2 h (B) postimmunization. Lethality was monitored daily for up to 10 days postchallenge. Kaplan-Meier plots show combined results of two independent experiments with a total of 10 mice/group and *P* values calculated by means of the log rank (Mantel-Cox) test.

The d-galactosamine (GalN)-sensitized mouse model of endotoxemia was used to assess the LPS-neutralizing potential of A1124 *in vivo* (Fig. 6). Mice were passively immunized with the A1124 MAb and 24 h later sensitized with GalN and challenged with lethal doses of purified LPS extracted from an ST131 strain. While animals receiving an isotype-matched control MAb rapidly succumbed to endotoxemia, A1124 afforded protection in a dose-dependent manner (Fig. 6A). In order to clarify whether the protection induced by A1124 was Fc dependent, the antibody F(ab′)_2_ and F(ab′) fragments of A1124 were tested in a subsequent experiment. Since the half-lives of the F(ab′)_2_ and F(ab′) fragments are expected to be less than 24 h and a few hours, respectively, the time interval between immunization and challenge was shortened to 2 h. In order to keep the number of antigen-binding sites constant, the efficacy of 6.6 μg of the fragments was compared to that of 10 μg of whole IgG molecules (Fig. 6B). The significant protection elicited by the IgG was abolished when using F(ab′)_2_ or F(ab′) fragments, suggesting that the Fc portion plays an indispensable role in the antibody-mediated protection against endotoxemia.

### Protection in rodent models of E. coli bacteremia.

**FIG 7:**
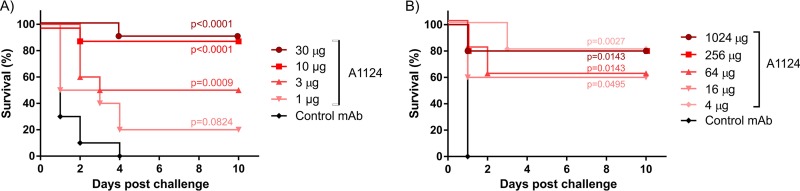
Protective efficacy of A1124 in rodent models of bacteremia. Animals were immunized with the indicated doses of MAbs and subsequently challenged intravenously with a lethal dose of E. coli ST131. (A) Murine model with a total of 10 mice per group (two identical experiments with 5 mice each) challenged with strain 81009. (B) Rat model with 5 animals per group challenged with strain 3O. In both models, animals were observed daily for 10 days postinfection. Differences in survival between the A1124- and the control MAb-treated groups were statistically compared by means of the log rank (Mantel-Cox) test with *P* values indicated.

The protective efficacy of A1124 was also tested *in vivo* in mouse and rat models of bacteremia using live E. coli ST131 challenge strains. Mice or rats passively immunized with serial dilutions of A1124 or an irrelevant control MAb were subsequently challenged intravenously with a lethal dose of live E. coli ST131 (minimal lethal doses determined in separate experiments [data not shown]). In both rodent species, significant protection was elicited at low doses of A1124 (Fig. 7). In the mouse model, a MAb dose-dependent protective effect was observed for A1124 in a dose range of 1 to 30 μg/animal (corresponding to approximately 50 to 1,500 μg/kg of body weight), where a dose of 10 μg/animal (approximately 0.5 mg/kg) elicited 90% protection (Fig. 7A). In rats, no MAb dose dependency could be determined, as all groups except the one treated with control MAb displayed 60 to 80% survival, and even the smallest amount of A1124 (4 μg/animal, corresponding to approximately 20 μg/kg) was significantly protective (Fig. 7B).

### Dissecting the modes of action for MAb-mediated protection.

**FIG 8:**
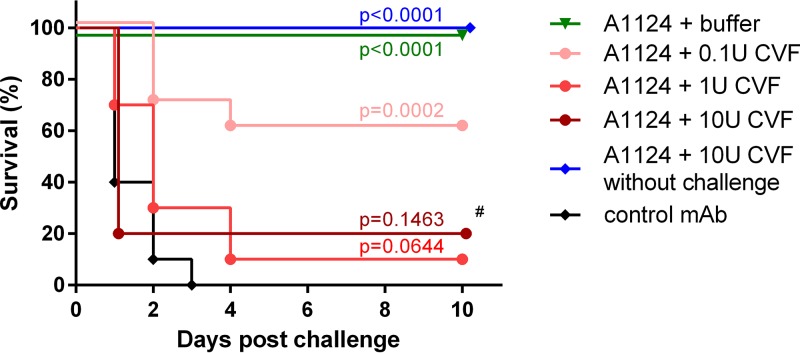
The role of the complement system in antibody-mediated protection in the murine model of bacteremia. Groups of 5 mice were dosed with the indicated amounts of cobra venom factor (CVF) or its buffer (control group) and subsequently passively immunized with 10 μg of A1124 or an irrelevant control MAb (control group). The next day, the animals were challenged intravenously with a lethal dose of E. coli ST131 strain 81009. Survival was monitored for up to 10 days postchallenge. Kaplan-Meier curves obtained from two independent experiments (with a total of 10 mice per group) are shown, except for the group marked with # (one experiment with *n* = 5 mice), and *P* values were calculated by means of the log rank (Mantel-Cox) test.

Since A1124 exhibits multiple effector functions *in vitro*, we wanted to elucidate which of these mechanisms contribute to protection in a bacteremia model. Therefore, the efficacy of A1124 was tested in mice treated with CVF to inhibit the complement activity (26). CVF had no effect on the survival of mice in the absence of bacterial challenge. Upon challenge, however, CVF (at the recommended concentration of 1 U or higher) abolished the antibody-mediated rescue, confirming that complement-dependent activities were indispensable for protection mediated by A1124 against lethal bacteremia caused by E. coli ST131 (Fig. 8).

## DISCUSSION

Consideration of antibacterial monoclonal antibodies for treating serious infections is facilitated by the emergence of multidrug-resistant bacteria. While in the case of toxin-mediated diseases (e.g., tetanus, botulism, diphtheria, Clostridium difficile infection, etc.) the selection of molecular targets is straightforward, designing effective antibodies against bacteria with a pathomechanism not relying on exotoxins is more challenging. Gram-negative bacteria can be contained by bactericidal antibodies that bind to their surface and activate the complement system initiated by C1q leading to the assembly of the membrane-attack complex (MAC). However, conserved surface antigens of Gram-negative bacteria tend to be masked by highly variable, mainly carbohydrate, structures that severely limit accessibility for antibody binding. On the other hand, targeting the often extensively variable outer polysaccharide antigens makes a broad-spectrum immunization approach difficult. Nevertheless, if a limited number of specific serogroups dominates among a clinically important class of pathogens, a precision targeted immunization approach against the serotype-determining surface polysaccharides may be appropriate. For instance, some successful clonal lineages (so-called “high-risk clones,” e.g., Klebsiella pneumoniae ST258 or E. coli ST131) are responsible for a significant proportion of MDR enterobacterial infections. While these clones appear to express different capsular types, the LPS O antigen is shared within the clonal lineage. Accessibility of the conserved LPS O antigen was proven in the cases of E. coli ST131 (24, 25) and K. pneumoniae ST258 (27) lineages, thereby providing attractive targets for immunization.

Antibodies against LPS O antigens have long been recognized to be protective. Bactericidal activities associated with O-antigen-specific antibodies include complement-mediated killing and opsonophagocytic killing. For both mechanisms, accessibility and density of the antigen are crucial for the simultaneous binding by adjacent C1q arms or Fc receptors, thereby validating the LPS O antigen as an attractive molecular target. Another mechanism of action of O-antigen-specific MAbs is the neutralization of the endotoxin activity of LPS. Although recognizing the carbohydrate part of LPS, O-antigen binding was reported to neutralize endotoxin activity associated with the lipid A portion in the case of several MAbs (28–30) and O-type-specific immune serum (31, 32). Finally, antivirulence activities, such as motility arrest (33, 34), were reported upon binding of MAbs to O antigens of different enterobacterial pathogens.

In this study, we show that a single MAb targeting the O25b antigen of the epidemic E. coli clone ST131-*H*30 is able to elicit multiple modes of action. Complement-dependent bactericidal activity was shown earlier (25) for a sibling humanized MAb (same CDR sequences with different humanized framework regions) and confirmed for A1124 in this study. This effect is not entirely unexpected in light of the abundant binding of O25b-specific MAbs to the surface of ST131 strains, irrespective of the type of the polysaccharide capsules (25). The high-density binding of the antibody allows efficient activation of C1q through the Fc part of the MAb, leading to the activation of the classical complement pathway and finally to the insertion of the membrane-attack complex (MAC) into the outer membrane of bacteria. This activity of A1124 can also be proven in a serum-free environment with *in vitro* reconstitution of the 9 complement factors involved in the classical pathway (M. Mutti, unpublished data). Upon tight binding onto the surface of E. coli, complement factors can bind to complement receptors (CRs) on the surface of phagocytes, leading to engulfment of bacteria. Alternatively, opsonophagocytosis can be mediated by Fcγ receptors (FcRs) that bind high-density antigen-bound IgG Fc portions. We showed that phagocytosis of E. coli ST131 by murine macrophages is significantly increased in the presence of MAb A1124 and active complement. Heat-inactivated complement did not support MAb-induced phagocytosis, suggesting that the observed uptake by RAW 264.7 cells was mediated entirely by the phagocyte complement receptors. Considering that the RAW 264.7 cell line is known to highly express Fcγ receptors and that the Fc portion of human IgG is readily bound by murine Fc receptors (35), the lack of FcR-mediated uptake is certainly surprising. The contribution of FcR- versus CR-mediated uptake may be affected by the relative abundance and surface localization, i.e., the proximity of IgG as well as complement binding relative to the membrane.

Both complement-mediated killing and opsonophagocytic killing were confirmed in a whole-blood assay that represents the most “*in vivo*-like” condition. The effect of the A1124 MAb was investigated in human blood in the presence of preexisting serum antibodies, human complement, and effector cells. Bactericidal activities triggered by A1124 could be observed in addition to the baseline bactericidal mechanisms of the human blood. Furthermore, the net killing induced by the MAb, which is the sum of complement-mediated killing and opsonophagocytic killing, could further be dissected by the addition of cytochalasin D (CytD), a potent inhibitor of actin polymerization and consequently phagocytosis. The lack of effect of CytD on the complement activity was proven in pilot experiments, thereby validating the assay. A similar assay was recently also proposed by another group for the determination of bacterial and host factors in the pathogenesis of bacteremia (36). In the characterization of biologics, this or similar high-throughput whole-blood assays could replace existing *in vitro* assays, which use serum and immortalized phagocyte cell lines, in order to translate to the clinical scenario as closely as possible.

The third mode of action that we were able to attribute to MAb A1124 was its endotoxin-neutralizing potency. Given that the antibody is specific to the O-antigen portion of LPS, which is separated from lipid A (the endotoxin) by the core oligosaccharide, it is unlikely that this activity is true neutralization, i.e., physical inhibition of the binding of lipid A to its innate receptor. This is also corroborated by the indispensability of the Fc part of A1124 in protection in the murine endotoxemia model that we utilized. Since LPS molecules are not expected to be available as single molecules but are rather associated with outer membrane components or form micelles (in the case of extracted purified LPS) (37), the observed “neutralization” potency is likely to originate from immune complex formation and Fc-dependent removal of these LPS-containing particles from the circulation as previously also suggested by others (38). Interestingly, the contribution of the Fc part was also proven in the case of MAbs that neutralize proteinaceous exotoxins, such as the anthrax toxins (39); hence, this mechanism for neutralization may be a more common phenomenon than currently appreciated. We could also corroborate the neutralizing potency of A1124 *in vitro* using an hTLR4 reporter assay as well as by inhibition of LPS-induced proinflammatory cytokine release from cultured macrophages. In these systems, the MAb activity may rely on alteration of micelle equilibrium, steric hindrance, and/or precipitation of micelles that may be independent of the Fc-dependent functions. Importantly, this activity was confirmed in the presence of human serum, where naturally occurring LPS binding proteins and preexisting antibodies might compete with A1124 for the binding of LPS molecules. Recently, we reported a MAb specific to the K. pneumoniae ST258 O antigen that outcompetes endotoxin neutralization potency of polymyxin B (an antibiotic that binds endotoxin stoichiometrically), by 3 orders of magnitude (28). This observation also supports a model in which O-antigen-specific MAbs act on the supramolecular structure of LPS. Further studies are needed, however, to elucidate the exact mechanism of endotoxin neutralization mediated by O-antigen-specific MAbs and the impact of neutralization on the protective efficacy of these MAbs in different animal models (40).

We tested the protective efficacy for A1124 in two rodent models of bacteremia. In both mice and rats, A1124 showed high levels of protection at very low antibody doses, applied prophylactically. Previously, we reported greatly reduced (but not fully abolished) efficacy of a sibling MAb of A1124 upon mutating the glycosylation site in the Fc region (25). This suggested that Fc-dependent mechanisms of action may be crucial for protection in the bacteremia model. However, as aglycosylated MAbs retain low-level complement-activating potency (41, 42), the exact contribution of the complement system could not be proven indisputably. In this study, the indispensability of the complement system was shown, since the MAb-afforded protection was lost in complement-depleted (cobra venom factor-treated) mice. In light of the complement dependency of phagocytosis (see above), this confirms that bactericidal activities are crucial to hinder development of lethal septicemia in this model. Still, it cannot be ruled out that the endotoxin neutralization activity has a potential additive effect to bactericidal mechanisms.

In summary, this study showed high prophylactic efficacy of A1124, a humanized monoclonal IgG targeting the O25b O antigen of E. coli ST131-*H*30. High-risk patients, such as immunocompromised or mechanically ventilated patients and those undergoing abdominal surgery, who are colonized with MDR ST131-*H*30 could benefit from O25b-specific MAbs in order to impede subsequent invasive infections. Since O25b is a conserved antigen within the ST131-*H*30 clone, this approach would currently cover 10 to 25% of all ExPEC strains and more than 50% of MDR isolates. In light of the high incidence of ExPEC infections (225,000 bacteremia cases and 150,000 intra-abdominal infections annually in the United States alone), a significant number of patients could benefit from this clone-specific approach. Prophylactic passive immunization may complement or replace standard prophylaxis by antibiotics. The low protective doses reported in this study may translate to lower production costs, which will allow a broad prophylaxis, analogous to vaccination but with the advantage of an immediate protective effect.

The patients colonized or infected with ST131-*H*30 could be easily identified by a rapid diagnostic assay. Currently, a PCR-based approach serves as the standard diagnostic tool to identify this clone by detecting an O25b-specific gene. As an alternative, a phenotypic assay utilizing O25b-specific MAbs was also validated for rapid detection (24).

The precision targeting of MAbs such as A1124 is expected to restrict collateral damage of the microbiota, an obvious advantage over antibiotic prophylaxis in most cases. Moreover, given the importance of the target antigen in virulence/fitness, the spread of escape mutants is unlikely. Based on these considerations, O25b-specific MAbs are attractive clinical candidates.

## MATERIALS AND METHODS

### Complement-mediated killing.

Serum bactericidal assays were performed as previously described (25). Briefly, a pool of human sera was incubated with bacteria (3 × 10^8^ CFU/ml) to remove specific antibodies, filter sterilized, and stored at −80°C. Complement- and antibody-mediated killing was assessed by incubating bacteria (3 × 10^4^ CFU/ml) in 50% adsorbed serum complemented with different concentrations of the test antibodies for 3 h. For CFU determination, the reaction mixtures were diluted in Luria-Bertani (LB) medium supplemented with 0.025% Tween 20 and plated on tryptic soy agar (TSA) plates (bioMérieux). Bacterial survival was expressed relative to the initial inoculum.

### Opsonophagocytic uptake.

The murine macrophage cell-line RAW 264.7 (ATCC TIB-71) was seeded in 96-well plates at a density of 7.5 × 10^4^ cells/well in Dulbecco's modified Eagle's medium (DMEM) (Invitrogen) supplemented with heat-inactivated 10% fetal bovine serum (FBS) and penicillin-streptomycin. The next day, the cells were washed twice with PBS and infected at an MOI of 1 with mid-log-phase-grown bacteria that were preopsonized for 15 min on ice in antibiotic-free medium supplemented with 2.5 μg/ml of the respective MAb and 5% active or heat-inactivated, adsorbed human serum. Cells and bacteria were incubated (1 h, 37°C), kanamycin was added to a final concentration of 40 μg/ml, and the mixtures were further incubated (2 h, 37°C) to eliminate the extracellular bacteria. Cells were subsequently washed twice with phosphate-buffered saline (PBS) and lysed with Triton-X (0.1%) for quantification of intracellular bacteria by plating and incubation (16 h, 37°C) of serial dilutions on TSA plates.

### Bactericidal effect in human blood.

The whole-blood assay was performed in 96-well plates. Eighty microliters of heparinized, human whole blood was mixed with 5 μl of 0.2 mg/ml cytochalasin D (Sigma-Aldrich) or buffer control and incubated on ice for 15 min. Antibodies and bacteria were added to a final concentration of 2.5 μg/ml and 2.5 × 10^4^ CFU/ml, respectively. The reaction mixture was incubated with mild shaking at 37°C for 3 h, and bacterial survival was quantified by plating and incubation (16 h, 37°C) of serial dilutions of the reaction mixture on TSA plates.

### Neutralization of LPS signaling.

LPS used in the *in vitro* neutralization assay was isolated by the hot phenol-water method as described in reference 24. Antibodies serially diluted in HEK Blue detection medium (InvivoGen), and where indicated with 4% human serum (prepared as a pool from three healthy donors at Red Cross Blutzentrale, Linz, Austria), were preincubated with 4 ng/ml of purified LPS for 30 min with shaking at room temperature in a volume of 50 μl. The reaction mixture was added to 50 μl hTLR4 reporter HEK Blue cell line (InvivoGen) resuspended in detection medium for a final concentration of 10^6^ cells/ml. After overnight incubation at 37°C in the presence of 5% CO_2_, absorbance was measured at 630 nm according to the manufacturer's instructions.

Antibody-mediated LPS neutralization was also analyzed using RAW 264.7 cells. Briefly, 7.5 × 10^4^ cells resuspended in 100 μl DMEM supplemented with 10% fetal calf serum (FCS) and penicillin-streptomycin were seeded in a 96-well plate and incubated overnight. The following day, cells were washed twice with PBS and a fresh 180-μl amount of antibiotic- and serum-free DMEM was added per well. Ten microliters of antibodies was added for a final concentration of 1.6 to 100 μg/ml. Purified LPS was diluted in DMEM and added at a final concentration of 10 ng/ml. The reaction mixture was incubated for 24 h, and the supernatants were collected and stored at −80°C for subsequent cytokine analyses. TNF-α and IL-6 levels were determined by enzyme-linked immunosorbent assay (ELISA) according to the manufacturer's instructions (Ready SET-Go; eBioscience, Vienna, Austria).

### Animal models.

Animal experiments were reviewed and approved by the Arsanis Animal Welfare and Ethics Committee and performed according to Austrian law (BGBl. I Nr. 114/2012) as approved by the respective competent authority (Magistratsabteilung 58, Vienna, Austria). In all experiments, groups of 5 female, 6- to 8-week-old BALB/cJRj mice (∼20 g in weight) were used (Janvier, France). The protective efficacy of monoclonal antibodies was assessed by intraperitoneal injection of serially diluted MAbs formulated in PBS, 24 h (or 2 h when testing antibody fragments) prior to challenge. Control groups received an isotype-matched (human IgG1) irrelevant control MAb at the highest dose. In the endotoxemia model, mice were sensitized by intraperitoneal administration of d-galactosamine (GalN; 20 mg/animal) and immediately challenged intravenously with LPS (2 ng) extracted from E. coli ST131 strain 81009 by using a commercial LPS extraction kit (Intron). In the bacteremia model, mice were intravenously challenged with 10^8^ CFU of a mid-log-phase-grown washed culture of strain 81009. Where indicated, complement was depleted by the intravenous administration of cobra venom factor (CVF; at the indicated doses) 24 h prior to lethal, intravenous challenge.

The rat model of bacteremia was performed at Fidelta Ltd. (Zagreb, Croatia) under institutional approval. Groups of five 9-week-old female Sprague-Dawley rats (Crl:SD; Charles River, France) (∼200 g in body weight) were intravenously immunized with serially diluted doses of MAb A1124 or an irrelevant control MAb. The next day, animals were intravenously challenged with a minimal lethal dose (10^9^ CFU/animal) of ST131 strain 3O.

All animals were housed under specific-pathogen-free (SPF) conditions, with food and water provided *ad libitum*. In all cases, survival was monitored daily for up to 10 days postchallenge. Statistical analyses were performed with the log rank (Mantel-Cox) test using GraphPad Prism 5.04 Software. Differences were considered statistically significant when *P* was <0.05.

## Supplementary Material

Supplemental material
