# Supplementary material for: Multiple Modes of Action of a Monoclonal Antibody against Multidrug-Resistant Escherichia coli Sequence Type 131-H30
Source: Antimicrob Agents Chemother. 2017 Oct 24;61(11):e01428-17. doi: 10.1128/AAC.01428-17 (PMC5655088; doi:10.1128/AAC.01428-17)
Supplement: Supplemental material [file supp_61_11_e01428-17__index.html]

Supplemental material 

# Multiple Modes of Action of a Monoclonal Antibody against Multidrug-Resistant Escherichia coli Sequence Type 131-*H*30

## Supplemental material

- Supplemental file 1 -

  Fig. S1 and S2

  PDF, 963K
