# Supplementary material for: Multiple Modes of Action of a Monoclonal Antibody against Multidrug-Resistant Escherichia coli Sequence Type 131-H30
Source: Antimicrob Agents Chemother. 2017 Oct 24;61(11):e01428-17. doi: 10.1128/AAC.01428-17 (PMC5655088; doi:10.1128/AAC.01428-17)
Supplement: Supplemental material [file AAC.01428-17_zac011176647s1.pdf]

Fig. S1

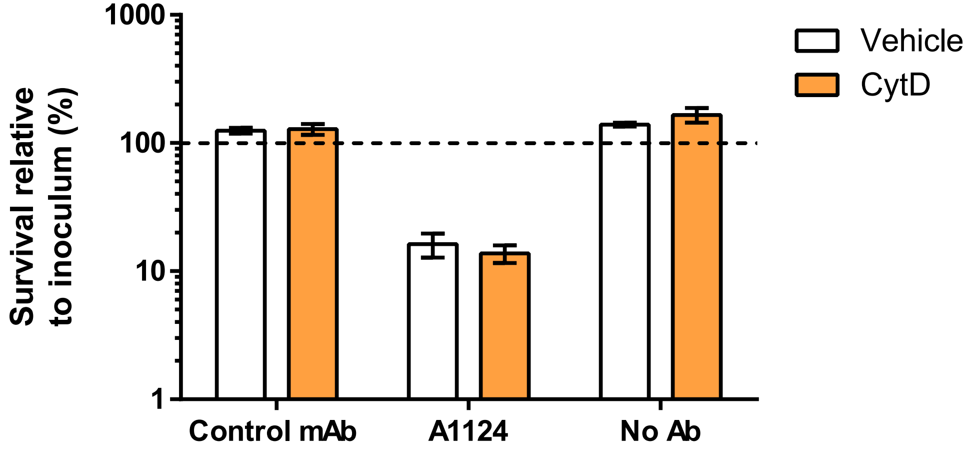


**Fig. S1. Effect of cytochalasin D on antibody/complement mediated bactericidal action.** Strain 81009 was incubated in 50% human plasma and 2.5 µg/mL of the test antibodies in the absence or presence of 10 µg/mL of cytochalasin D for 3 hours.

Fig. S2

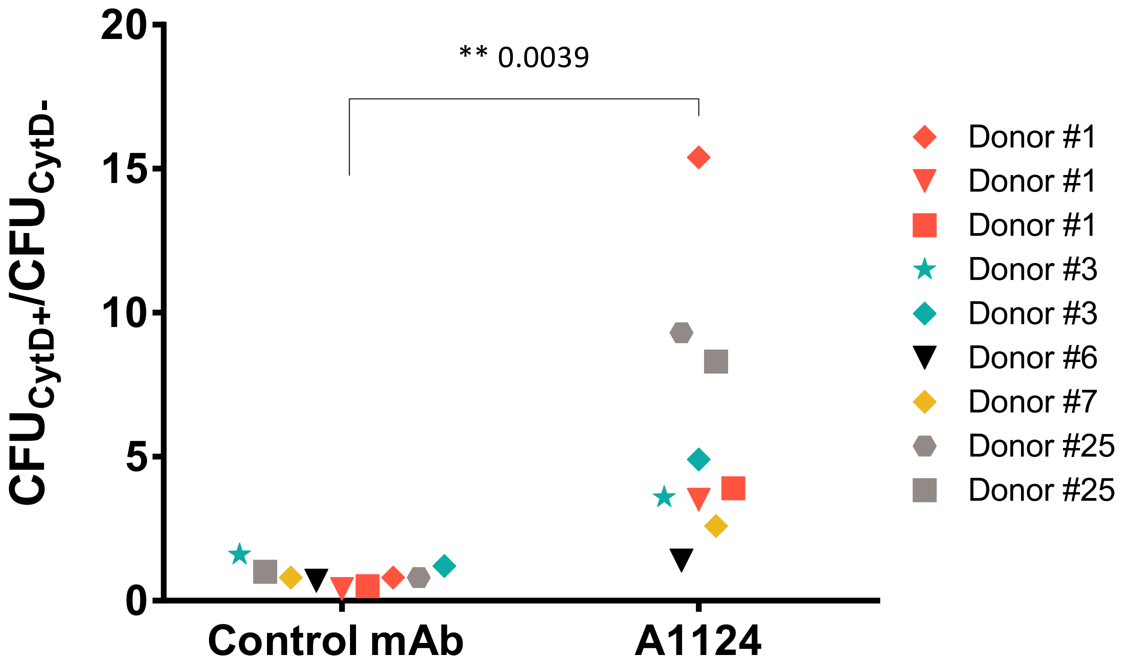


**Fig. S2. Dissection of phagocyte dependent and independent bactericidal action of human whole blood in the presence of A1124.** The ratio of surviving bacterial count in whole blood treated with cytochalasin D or a control buffer was calculated in presence of A1124 or an isotype control mAb. Ratio 1 indicates that no phagocyte function was involved in the bactericidal action of whole blood. Graph shows combined results of 9 experiments, statistical comparison was performed with Wilcoxon matched-pairs signed rank test.
